# Alcohol-related mortality among adults

**DOI:** 10.17886/RKI-GBE-2016-028

**Published:** 2016-09-28

**Authors:** Alexander Rommel, Anke-Christine Saß, Martina Rabenberg

**Affiliations:** Robert Koch Institute, Department for Epidemiology and Health Monitoring, Berlin, Germany

**Keywords:** ALCOHOL DEPENDENCY, ALCOHOL ABUSE, MORTALITY, ADULTS

## Abstract

Risky, abusive and addictive consumption of alcoholic beverages greatly jeopardizes the drinker’s health; it can harm third parties, affects the drinker’s social relations and causes considerable costs to the national economy. National and international campaigns therefore aim to lower alcohol consumption and its consequences. Alcohol-related mortality is the most serious outcome of excessive alcohol consumption. Official statistics on causes of death include a number of entirely alcohol-related diagnoses. In 2014 an examination of deceased adults in Germany revealed a directly alcohol-related cause of death in 14,095 cases (20.8 for every 100,000 inhabitants). Such diagnoses were considerably more frequent for men than women, reaching a peak in the 55 to 64 age group. Overall, death from alcohol abuse is declining in Germany. Because by international standards there is still a relatively high consumption of alcoholic beverages in Germany, more action needs to be taken.

## Introduction

Risky, abusive and addictive consumption of alcoholic beverages greatly jeopardizes the drinker’s health; it can harm third parties, affects social relations and causes considerable costs to the national economy [1]. The prevention of alcohol-related problems is thus an important part of many public health strategies. The WHO’s “Global action plan for the prevention and control of non-communicable diseases 2013-2020” calls for a relative reduction of high-risk alcohol consumption by 10% between the years 2010 and 2025 [2]. In Germany the battle against alcohol consumption and its consequences has been included in the list of National Health Objectives. Here too the aim is to lower the amount of alcohol consumed by Germans [3].

The permanent monitoring of key indicators is vital in order to estimate the extent to which the objectives have been attained. Accordingly, the World Health Organization (WHO) proposes a campaign to measure the per capita consumption of pure alcohol, the prevalence of severe binge-drinking and alcohol-related morbidity and mortality, which would be taken as key indicators; it also recommends expanding this list to fit the national context. In this edition of the Journal of Health Monitoring information is provided about risky alcohol consumption and per capita intake in the Focus Alcohol consumption of adults in Germany: Harmful drinking quantities, consequences and measures. There are also Fact sheets on Cases of alcohol poisoning involving in-patient treatment and Traffic accidents under the influence of alcohol. This Fact sheet complements the information contained in the aforesaid with details of alcohol-related mortality - the most severe result of excessive alcohol consumption.

## Indicator

The indicators on fatalities are taken from the cause of death statistics, which represent a complete annual record of all deaths in Germany. This is based on the doctors’ death certificates listing the diseases which have led to the person’s death. In accordance with the WHO code the cause of death statistics follows a monocausal approach i.e. only the underlying illness is extracted from the entries in the death certificate for inclusion in the statistics. Since January 1st, 1998, the 10th Revision of the “International Statistical Classification of Diseases and Related Health Problems” (ICD-10) has been applied when preparing the official cause of death statistics. To determine the alcohol-related deaths, reference is made to a list of diseases caused in their entirety by alcohol [4]. The diagnoses F10 (mental and behavioural disorders due to alcohol) and K70 (alcoholic liver disease) are identified as responsible for more than 90% of these directly alcohol-related deaths and will be shown separately in the following [5, 6]. Causes of death such as cardiovascular disease or cancers which may have been developed in large part due to excessive consumption of alcohol are not included in the following analyses. Only those deaths caused entirely by alcohol are analysed by age, gender and year. The overall mortality attributable to alcohol is thus underestimated. The report encompasses absolute numbers of deaths and deaths per 100,000 of the population over the age of 18. Age standardized figures for comparisons over time are compiled using the “old” European Standard Population and relate to all age groups from the age of 0 years.

## Classification of findings

In 2014, the cause of death for a total of 14,095 adults in Germany was ascertained to be an entirely alcohol-related disease. Thus 20.8 of 100,000 inhabitants over the age of 18 died of a disease directly associated with the consumption of alcohol. Considerably more men than women die of causes attributable to alcohol (Table 1). Men thus account for almost three quarters of such deaths.

**Fig. 1:**
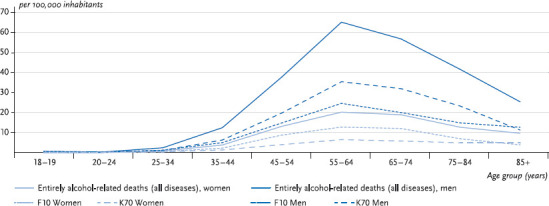
Alcohol-related mortality among adults by age, 2014 (deaths per 100,000 inhabitants) Source: Cause of death statistics [6]

In regional terms, the prevalence of alcohol-related mortality is well above average in the “new” eastern Länder and in Bremen [6]. Moreover, mortality due to alcohol-induced disorders strongly correlates with age. Overall, and in terms of the major specific diagnoses, a notable rise in deaths first occurs in the 35 to 44 age group. Thereafter directly alcohol-related mortality increases sharply until it reaches a peak in the age group 55 to 64. Here the alcohol-related mortality is 20.2 per 100,000 inhabitants for women and 65.4 per 100,000 inhabitants for men. Thus, in parallel with the increased mortality due to age, the gender gap to the disadvantage of men successively evolves with advancing age. In the younger age groups it is seen that there is little difference between men and women in terms of alcohol-related mortality (Fig. 1).

The gender-specific distribution of alcohol-related causes of death is mirrored in the patterns of consumption that become established in early adulthood: Risky drinking behaviour and binge drinking are appreciably more prevalent among men than women [7, 8] (Focus). With increasing age, some consumption patterns such as binge drinking decline among both genders [7], but some people display a chronic pattern of alcohol abuse. The comparatively high alcohol-related mortality seen in middle age is thus due to the fact that these people have accumulated risks for disease and death over a longer period of their lives.

Similarly, the regional differences in alcohol-related mortality correspond with differing patterns of consumption: since the fall of the Wall and up to this day, alcohol consumption in the “new” Länder has been higher than that in the “old” Länder. However, barely any difference is now ascertainable in the youth and young adult age groups [9]. There is therefore reason to hope that the trend towards a lessening of the east-west divide in alcohol-related mortality rates will continue [6].

Over the course of time there has been a decline in entirely alcohol-related mortality in Germany [6]. In respect of all age groups (from 0 years) the age standardized rates for men have fallen from 29.1 deaths per 100,000 inhabitants in 1998 to 20.1 in 2014. Starting from a lower base, the figures for women showed a slower decline from 9.0 deaths to 6.5 per 100,000.

The decline in alcohol-related mortality over time matches patterns of lower consumption: various studies come to the same conclusion that over the last thirty years the proportion of people who drink dangerous amounts of alcohol has fallen [10] (Focus). Fundamentally, therefore, there are positive developments in Germany that mark progress towards the national and international targets. Since, however, Germany still falls into the one quarter of OECD member states registering the highest per capita consumption of alcohol, and its consumption is also relatively high in global terms, action is still required [11, 12]. Possible approaches range from steps to provide information and education that appeal directly to the consumers, to structural measures, by taking a closer look at advertising for alcoholic drinks, pricing policies and the availability of alcohol (Focus). Programmes offered by addiction care services are very important aids to avoiding alcohol-induced mortality, as they help recognize and treat alcohol dependency in time. Since alcohol consumption and alcohol-related mortality are phenomena that manifest disproportionally in men, prevention and therapy need to take a gender sensitive approach in order to make an adequate response to the specific behavioural patterns of men and women [13].

## Key statements

- In 2014, 14,095 adults died from a directly alcohol-related disease.
- Men account for about three quarters of all deaths directly attributable to alcohol.
- Starting in the 35 to 44 age group alcohol-related mortality increases sharply until it reaches a peak in the age group 55 to 64.

## Figures and Tables

**Table 1:** Alcohol-related mortality among Adults in 2014 (≥age 18) Source: Cause of death statistics [6]

		Deaths	Deaths per 100,000 inhabitants (raw data)
**K70 Alcoholic liver disease**	Total Women Men	7,864 2,256 5,608	11.6 6.5 17.0
**F10 Mental and behavioural disorders due to alcohol**	Total Women Men	5,113 1,192 3,921	7.5 3.4 11.9
**Entirely alcohol-related deaths (all diseases)**	Total Women Men	14,095 3,672 10,423	20.8 10.5 31.6
